# Influence of astragaloside IV on pharmacokinetics of triptolide in rats and its potential mechanism

**DOI:** 10.1080/13880209.2019.1702705

**Published:** 2020-04-01

**Authors:** Jian Gao, Xiangmin Zeng, Wei Zhao, Desheng Chen, Jing Liu, Ning Zhang, Xingliang Duan

**Affiliations:** a Department of Image, Yidu Central Hospital of Weifang, Shandong, China; b Department of Ultrasonography, Yidu Central Hospital of Weifang, Shandong, China; c Department of Pediatric Medicine, Yidu Central Hospital of Weifang, Shandong, China; d Department of Emergency, Weifang People’s Hospital, Shandong, China

**Keywords:** Caco-2 cells, drug–drug interaction, oral absorption, P-gp

## Abstract

**Context:**

It is common to combine two or more drugs in clinics in China. Triptolide (TP) has been used primarily for the treatment of inflammatory and autoimmune diseases. Astragaloside IV (AS-IV) has been applied with many other drugs, due to its various pharmacological effects. AS-IV and TP can be used together for the treatment of diseases in clinics in China.

**Objective:**

This study investigates the effects of astragaloside IV (AS-IV) on the pharmacokinetics of TP in rats and its potential mechanism.

**Materials and methods:**

The pharmacokinetics of orally administered triptolide (2 mg/kg) with or without AS-IV pre-treatment (100 mg/kg/day for 7 d) were investigated. Additionally, the effects of AS-IV on the transport of triptolide were investigated using the Caco-2 cell transwell model.

**Results:**

The results indicated that when the rats were pre-treated with AS-IV, the *C*_max_ of triptolide decreased from 418.78 ± 29.36 to 351.31 ± 38.88 ng/mL, and the AUC_0-t_ decreased from 358.83 ± 19.56 to 252.23 ± 15.75 μg/h/L. The Caco-2 cell transwell experiments indicated that AS-IV could increase the efflux ratio of TP from 2.37 to 2.91 through inducing the activity of *P-gp*.

**Discussion and conclusions:**

In conclusion, AS-IV could decrease the system exposure of triptolide when they are co-administered, and it might work through decreasing the absorption of triptolide by inducing the activity of *P-gp*.

## Introduction

Triptolide (TP), a diterpenoid triepoxide, is a major pharmacological component isolated from *Tripterygium wilfordii* Hook F (Celastraceae) (TWHF) (Brinker and Raskin 2005), and it has been used primarily for the treatment of inflammatory and autoimmune diseases such as rheumatoid arthritis, systemic lupus erythematosus, and skin diseases (Cheng et al. 2014; Li et al. 2014). Recently, research has reported that TP possessed a potent anti-cancer effect against gastric cancer, pancreatic cancer, and lung cancer (Liu et al. 2011; Ling et al. 2014; Park 2014). Based on its multiple biological activities and remarkable clinical performance, TP has attracted the attention of researchers and gained wide acceptance in the world in recent decades. However, the clinical application of TP was restricted because of its narrow therapeutic range and severe toxicity to digestive, reproductive and haematopoietic systems (Li et al. 2014; Singla and Challana 2014).

*Radix astragali* [the root of *Astragalus mongholicus* Bge. or *Astragalus membranaceus* Bge. (Fabaceae)] has been used as one of the primary tonic herbs in traditional Chinese and Japanese Kampo medicine (Deng et al. 2016; Zhao et al. 2017). Astragaloside IV (AS-IV) is one of the major active compounds of *Radix Astragali*. AS-IV possesses a number of pharmacological effects, including immunoregulatory, anti-hypertensive, antioxidative, anti-inflammatory and antitumor effect (Luo et al. 2004; Zhang et al. 2003, 2006, 2011; Li et al. 2016). Zhang et al. (2016) have reported that AS-IV could induce the activity of *P-gp*, which might lead to drug–drug interactions when they are co-administered with other herbs or drugs that are *P-gp* substrates. As we know, AS-IV and TP might be used together for the treatment of diseases in Chinese clinics, such as rheumatic diseases and diabetic nephropathy (Shi et al. 2016). In addition, many herb–drug interactions resulting from the concurrent use of herbal drugs with over-the-counter drugs may cause adverse reactions such as toxicity and treatment failure. However, the drug–drug interactions between AS-IV and TP are still unknown. Therefore, there is an urgent need to investigate the potential for drug-drug interactions of TP.

In this study, the potential drug-drug interactions of TP with AS-IV were systematically investigated. The *in vivo* pharmacokinetics of TP in rats with or without pre-treatment were investigated. The effects of AS-IV on the absorption of TP were investigated in the Caco-2 cell transwell model.

## Materials and methods

### Chemicals and reagents

TP (purity >98%) and AS-IV (purity >98%) were purchased from the National Institute for the Control of Pharmaceutical and Biological Products (Beijing, China). Dulbecco’s modified Eagle’s medium (DMEM) and non-essential amino acid (NEAA) solution were purchased from Thermo Scientific Corp. (Logan, UT). Foetal bovine serum (FBS) was obtained from GIBCO BRL (Grand Island, NY). Penicillin G (10,000 U/mL) and streptomycin (10 mg/mL) were purchased from Amresco (Solon, OH). Hanks' balanced salt solution (HBSS) was purchased from GIBCO (Grand Island, NY). Acetonitrile and methanol were purchased from Fisher Scientific (Fair Lawn, NJ). Formic acid was purchased from Anaqua Chemicals Supply Inc. Limited (Houston, TX). Ultrapure water was prepared with a Milli-Q water purification system (Millipore, Billerica, MA). All other chemicals were of analytical grade or better.

### Animal experiments

Male Sprague–Dawley (SD) rats weighing 220–250 g were provided by Sino-British Sippr/BK Lab Animal Ltd. (Shanghai, China). Rats were bred in a breeding room at 25 °C with 60 ± 5% humidity and a 12 h dark/light cycle. Tap water and normal chow were given *ad libitum*. All of the experimental animals were housed under the above conditions for a 3-day acclimation period and fasted overnight before the experiments. All experimental procedures and protocols were reviewed and approved by the Animal Care and Use Committee of Yidu Central Hospital of Weifang.

### *In vivo* pharmacokinetic study

The rats were divided into two groups of six animals each, including TP-only group (A) and AS-IV + TP group (B). TP (200 μg/mL) was dissolved in normal saline containing PEG400 (1:20) and orally administered to rats at a dose of 2 mg/kg. The test group was pre-treated with AS-IV at a dose of 100 mg/kg/day for 7 d before the administration of TP. Blood samples (250 μL) were collected into heparinised tubes via the *oculi chorioideae* vein at 0, 2, 10, 15, 30, 45, 60, 90, 120, 180, 240, 360, 480, and 600 min after the TP treatment, respectively. The blood samples were centrifuged at 5000 rpm for 5 min, and the plasma samples obtained were stored at −40 °C until the analysis.

### Preparation of rat plasma samples

To 100 μL aliquot of a plasma sample, 20 μL methanol and 180 μL internal standard methanol solution (2 ng/mL) were added and vortexed for 60 s to mix in a 1.5 mL polypropylene tube, and were centrifuged at 12,000 rpm for 10 min. The supernatant was removed into an injection vial, and a 3 μL aliquot was injected into the LC-MS/MS system for analysis.

### Instruments and conditions

The LC-MS/MS system was employed for the analysis of TP, according to previous studies (Xu et al. 2019). The analysis was performed on an Agilent 1290 series liquid chromatography system (Agilent Technologies, Palo Alto, CA), including a binary pump, an on-line vacuum degasser, a surveyor autosampling system, a column temperature controller, and an Agilent 6460 triple-quadruple mass spectrometer (Agilent Technologies, Palo Alto, CA) with Turbo Ion spray, which is connected to the liquid chromatography system. An Agilent MassHunter B 4.0 software was used for the control of equipment, data acquisition, and Agilent Quantitative analysis software was used for data analysis. The chromatographic analysis of TP was performed on a Shiseido MG-C18 column (3.0 × 100 mm, i.d.; 3.0 μm, Japan) at room temperature. The mobile phase was water (containing 0.1% formic acid) and acetonitrile (36: 64, v:v) at a flow rate of 0.6 mL/min, and the split ratio was 1:1.

The mass scan mode was positive MRM mode. The precursor ion and product ion are *m*/*z* 361.3→128.2 for TP, and *m*/*z* 363.5→121.0 for IS, respectively. The collision energy for TP and IS were 30 and 20 eV, respectively. The MS/MS conditions were optimised as follows: fragmentor, 110 V; capillary voltage, 3.5 kV; Nozzle voltage, 500 V; nebuliser gas pressure (N_2_), 40 psig; drying gas flow (N_2_), 10 L/min; gas temperature, 350 °C; sheath gas temperature, 400 °C; sheath gas flow, 11 L/min.

### Data analysis

The pharmacokinetic parameters, including the area under the plasma concentration-time curve (*AUC*), maximal plasma concentration (*C*_max_), the time for the maximal plasma concentration (*T*_max_), and the mean residence time (*MRT*) were calculated using the DAS 3.0 pharmacokinetic software (Chinese Pharmacological Association, Anhui, China).

The differences between the mean values were analyzed for significance using a one-way analysis of variance (ANOVA). Values of *p <* 0.05 were considered to be statistically significant.

### Cell culture

The Caco-2 cell line was obtained from the American Type Culture Collection (Manassas, VA). The Caco-2 cells were cultured in DMEM high glucose medium containing 15% FBS, 1% NEAA and 100 U/mL penicillin and streptomycin (Liu et al. 2018). The cells were cultured at 37 °C with 5% CO_2_. For transport studies, the cells at passage 40 were seeded on transwell polycarbonate insert filters (1.12 cm^2^ surface, 0.4 μm pore size, 12 mm diameter; Corning Co-star Corporation, Corning, MA) in 12-well plates at a density of 1 × 10^5^ cells/cm^2^. Cells were allowed to grow for 21 days. For the first 7 d, the medium was replaced every 2 d, and then daily. The transepithelial electrical resistance (TEER) of the monolayer cells was measured using Millicell ERS-2 (Millipore Corporation, Billerica, MA), and TEER exceeding 400 Ω.cm^2^ was used for the flux experiment. The integrity of the Caco-2 monolayers was confirmed by the paracellular flux of Lucifer yellow, which was less than 1% per hour. The alkaline phosphatase activity was validated using an Alkaline Phosphatase Assay Kit. The qualified monolayers were used for transport studies.

### Effects of as-IV on the absorption of TP in the caco-2 cell transwell model

Before the transport experiments, the cell monolayers were rinsed twice using warm (37 °C) Hanks’ balanced salt solution (HBSS), then the cells were incubated at 37 °C for 20 min. After preincubation, the cell monolayers were incubated with TP in fresh incubation medium added on either the apical or basolateral side for the indicated times at 37 °C. The volume of incubation medium on the apical and basolateral sides was 0.5 mL and 1.5 mL, respectively, and a 100 μL aliquot of the incubation solution was withdrawn at the indicated time points from the receiver compartment and replaced with the same volume of fresh pre-warmed HBSS buffer. The inhibitory effects of *P-gp* inhibitors on the TP flux by Caco-2 cells were investigated by adding 50 μM AS-IV to both sides of the cell monolayers and preincubating the sample at 37 °C for 30 min. The permeability of TP (10 μM) in all of the above conditions for both directions, i.e., from the apical (AP) side to the basolateral (BL) side and from the BL side to the AP side, was measured after incubation for 30, 60, 90 and 120 min at 37 °C. In addition, the efflux activity of *P-gp* was validated using a typical *P-gp* substrate digoxin (25 μM).

The apparent permeability coefficient (*P_app_*) was calculated using the equation of Artursson and Karlsson:

$$P_{\mathrm{app}}=\left(\Delta Q/\Delta t\right)\times \left[1/\left(A\times C_{0}\right)\right]$$

where *P_app_* is the apparent permeability coefficient (cm/s), ΔQ/Δt (μmol/s) is the rate at which the compound appears in the receiver chamber, *C_0_* (μmol/L) is the initial concentration of the compound in the donor chamber and A (cm^2^) represents the surface area of the cell monolayer. Data were collected from three separate experiments, and each was performed in triplicate.

## Results

### Effects of AS-IV on the pharmacokinetics of TP

**Figure 1.**
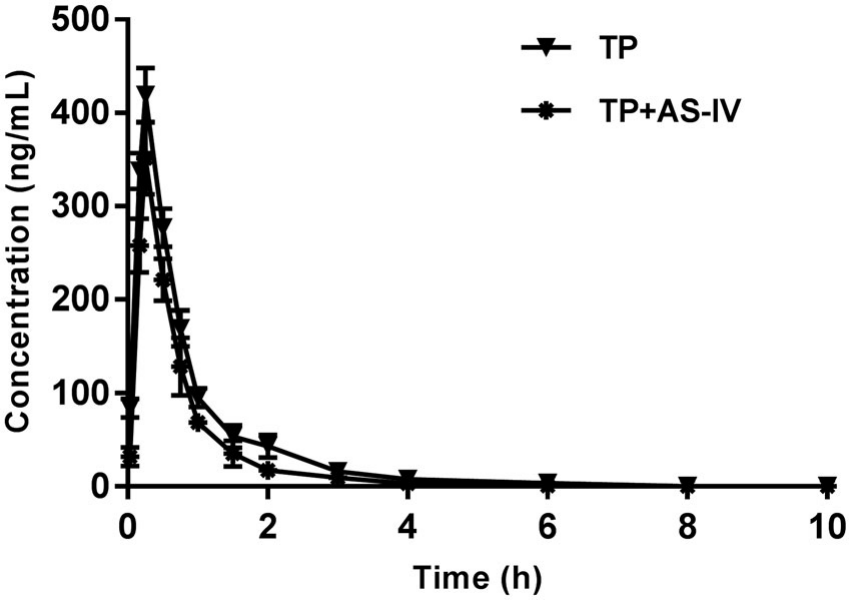
The pharmacokinetic profiles of triptolide in rats after the oral administration of 2 mg/kg triptolide with or without AS-IV pre-treatment.

The mean plasma concentration-time curves of TP after both the oral administration of TP and the oral administration of TP with AS-IV are presented in Figure 1.

The pharmacokinetic parameters of TP were calculated using the noncompartmental method with the DAS 3.0 pharmacokinetic software (Chinese Pharmacological Association, Anhui, China). The pharmacokinetic parameters are shown in Table 1.

**Table 1.** Pharmacokinetic parameters of triptolide in rats after oral administration of triptolide (2 mg/kg; *n* = 6, mean ± SD) with and without treatment of AS-IV.

Parameter	Control	Pre-treatment of AS-IV
*T*_max_ (h)	0.26 ± 0.08	0.23 ± 0.03
*C*_max_ (μg/L)	418.78 ± 29.36	351.31 ± 38.88
*t*_1/2_ (h)	0.45 ± 0.08	0.52 ± 0.12
AUC (0-t) (μg h L^−1^)	358.83 ± 19.56	252.23 ± 15.75*
CL (L/h/kg)	5.59 ± 0.31	7.95 ± 0.51*

*p < 0.05 indicates significant differences compared with the control.

As the results showed, pre-treatment with AS-IV (100 mg/kg/day for 7 d) in rats was associated with a significant decrease of AUC_0–t_ (decreased by approximately 30%) compared with the control (*p* < 0.05). The *C*_max_ decreased by approximately 16%, and this difference was not significant. The *t*_1/2_ of TP in rats pre-treated with AS-IV was shortened compared with the control, but this difference was not significant. Although the absorption of TP increased with co-administration of AS-IV, there was no change in *T*_max_.

### Effects of as-IV on the bidirectional transport of TP across caco-2 cells

**Figure 2.**
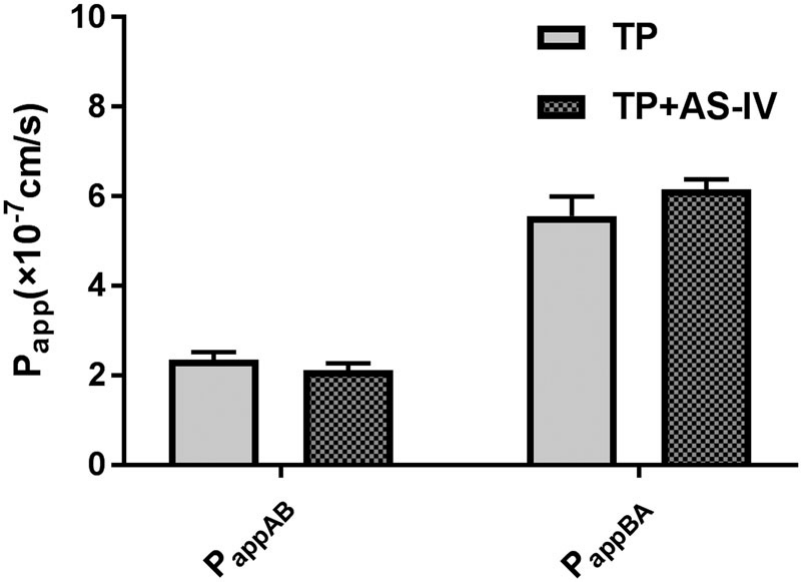
The effects of AS-IV on the bidirectional transport of triptolide from the apical to basolateral side (A) or in the opposite direction (B). Caco-2 cell monolayers were incubated at 37 °C in HBSS (pH 7.4), and triptolide (25 μM) was added to the donor chamber of the Caco-2 cell monolayer. Each point represents the mean ± SD of three determinations.

To validate the efflux activity of *P-gp*, a typical *P-gp* substrate digoxin was used, and the results indicated that the efflux ratio of digoxin was 12.1, which was abrogated in the presence of a typical *P-gp* inhibitor verapamil. The results indicated that the efflux activity of *P-gp* was qualified for the experiment. Then the transport of 10 μM of TP across Caco-2 cell monolayers was investigated in this study. As shown in Figure 2, the *P_appAB_* and *P_appBA_* were 2.35 ± 0.18 × 10^−7 ^cm/s and 5.57 ± 0.42 × 10^−7 ^cm/s, respectively. The *P_appBA_* was much higher than the *P_appAB_*, which indicated that efflux transporters might be involved in the transport of TP. Then, the transport studies were performed in the presence of AS-IV. As shown in Figure 2, in the presence of 50 μM of AS-IV, the *P_app_* values from the AP side to the BL side decreased, whereas those from the BL side to the AP side increased. The efflux ratio increased from 2.37 to 2.91, and the efflux of TP was increased. These results indicated that *P-gp* was involved in the transport of TP.

## Discussion

This is the first study to investigate the effects of AS-IV on the pharmacokinetics of TP in rats. Generally, to treat inflammation, autoimmune diseases or tumours, TP is mainly recommended for oral administration under professional guidance. So, in our pharmacokinetics study, TP was orally administered to rats at a dose of 2 mg/kg. The results indicated that AS-IV could decrease the systemic exposure of TP in rats when they were co-administered. The Caco-2 cell transwell experiments revealed that AS-IV could enhance the efflux ratio of TP, most probably, by inducing the activity of *P-gp*, as *P-gp* hinder its absorption in intestine.

Chinese medicines are often co-administered in clinical practice with or without patients’ knowledge, which may greatly raise the potential of drug–drug interactions. Drug-herb interactions may be mediated by physicochemical properties, pharmacokinetic properties and pharmacodynamic properties. Among them, pharmacokinetic properties are the main cause. Pharmacokinetic interaction between Chinese medicine and drug may occur in the phase of absorption, distribution, metabolism or excretion (Kim et al. 1998; Kataoka et al. 2011; Meng and Liu 2015).

As we know, AS-IV is usually used together with other herbs or medicines for the treatment of different diseases, Therefore, drug–drug interaction should be cautioned when AS-IV is co-administered with other herbs or drugs. Previous studies have shown that exposure to AS-IV induced the protein levels of *P-gp* in HepG2 cell line (Zhang et al. 2016), and we also found pre-treatment with AS-IV increased the efflux ratio of TP significantly. So, we infer that AS-IV lifted the efflux ratio of TP by inducing the activity of *P-gp*, as *P-gp* hinder absorption of TP in intestine.

This study also had some limitations. MRP transporters also expressed in Caco-2 cell, as AS-IV could not affect the activity of MRP transporters, and therefore, it was not investigated in this study. Excretion phase might also play an important role in the drug-drug interaction between AS-IV and TP, but it was not investigated in this study.

In conclusion, this study’s results indicate that when the rats were pre-treated with AS-IV, the system exposure of TP would be decreased significantly. This work offers a useful reference for the reasonable and safe co-administration of TP with clinically prescribed herbal or natural products which contain AS-IV, to reduce TP-induced toxicity.

## Conclusions

In this study, the effects of AS-IV on the pharmacokinetics of TP in rats were investigated, and the effects of AS-IV on the *in vitro* absorption of TP were also studied using the Caco-2 cell transwell model. The results indicated that AS-IV could significantly affect the pharmacokinetics of TP in rats, and the *in vitro* experiments indicated that AS-IV could decrease the absorption of TP. In conclusion, this study indicates that co-administration of TP with clinically prescribed herbal or natural products which contain AS-IV would decrease the system exposure of TP, and the dose of TP should be adjusted accordingly.
